# A Randomized, Open-Labeled, Prospective Controlled Study to Assess the Efficacy of Frontline Empirical Intravenous Piperacillin/Tazobactam Monotherapy in Comparison with Ceftazidime Plus Amikacin for Febrile Neutropenia in Pediatric Oncology Patients

**DOI:** 10.31557/APJCP.2019.20.9.2733

**Published:** 2019

**Authors:** Ruchirek Kamonrattana, Lalita Sathitsamitphong, Worawut Choeyprasert, Pimlak Charoenkwan, Rungrote Natesirinilkul, Kanda Fanhchaksai

**Affiliations:** 1 *Department of Pediatrics, Faculty of Medicine,*; 2 *Research Cluster of Thalassemia and Red Blood Cell Disorders, Faculty of Medicine, Chiang Mai University, Chiang Mai, Thailand.*

**Keywords:** Piperacillin/tazobactam, ceftazidime, amikacin, febrile neutropenia, children

## Abstract

**Background:**

Febrile neutropenia (FN) is the most common complication in pediatric oncology patients. Appropriate empirical antibiotics treatment is essential for treatment outcome.

**Methods:**

This study was a randomized prospective controlled study to demonstrate the efficacy of piperacillin/tazobactam (PIP/TZO) monotherapy compared with ceftazidime/amikacin in children with FN. Pediatric oncology patients at Chiang Mai University Hospital, diagnosed with FN, were randomized to receive either PIP/TZO 320 mg/kg/day divided every 8 hours or ceftazidime 100 mg/kg/day divided every 8 hours plus amikacin 15 mg/kg/day once daily. Treatment responses were compared between the two groups.

**Results:**

One-hundred and eighteen febrile neutropenic episodes in 70 patients (42 males and 28 females) were enrolled. The median age was 7 (3-10) years. The early response and complete response to initial treatment were achieved in 48/59 (81.4%) episodes and 41/59 (69.5%) episodes in PIP/TZO group compared with 40/59 (67.8%) episodes and 33/59 (55.9%) episodes in ceftazidime/amikacin group (*p-value* 0.091 and 0.128, respectively). Treatment modification in PIP/TZO group was required in 18/59 (30.5%) compared with 26/59 (44.1%) patients in ceftazidime/amikacin group (*p-value *0.128). Similarly, the duration of fever, duration of neutropenia and duration of antibiotics treatment were not significantly different between two groups. No serious adverse events were observed.

**Conclusion:**

The treatment responses of PIP/TZO monotherapy and ceftazidime/amikacin therapy were not significantly different. Both therapies were effective for FN in pediatric oncology patients.

## Introduction

Neutrophils are important parts of the immune system as they are the first defense mechanism against infections (Crawford et al., 2004). Febrile neutropenia (FN) is a common complication in pediatric oncology patients who received intensive cytotoxic chemotherapy. FN should be considered as urgent medical condition because delayed treatment results in morbidity and mortality in patients with cancer (Ichikawa et al., 2011). The reported prevalence of bacteremia was 47% in febrile neutropenic patients (Hakim et al., 2009). Prompt empirical antibiotics therapy is essential for patients with FN (Hess et al., 1998). In the last three decades, the mortality rate of FN improved from approximately 90% in early 1960s to less than 5% in 1990s due to the prompt empirical antibiotics and improvement of supportive care (Hess et al., 1998; De Pauw et al., 1994). The current reported mortality rate is 1-4% in pediatric patients with FN (Laoprasopwattana et al., 2007; Hann et al., 1997). 

Piperacillin/tazobactam (PIP/TZO) is a combination of board spectrum β-lactam antibiotic and a potent β-lactamase inhibitor which is appropriate for various infections (Gorschlüter et al., 2003). The antimicrobial spectrum of PIP/TZO is wide and include gram-positive aerobic organisms, gram-negative aerobic organisms and anaerobes. The common pathogenic organisms in FN including *Staphylococcus spp*, *Escherichia coli*, *Pseudomonas aeruginosa* or *Enterobacteriaceae* usually respond well to PIP/TZO (Ichikawa et al., 2011). The guidelines of the Infectious Disease Society of America (IDSA), published in 2010, recommended empirical antibiotics monotherapy with anti-pseudomonal β-lactam agents such as cefepime, carbapenem or PIP/TZO in high risk febrile neutropenic patients who required hospitalization. Other antibiotics, such as aminoglycosides, fluoroquinolones, and/or vancomycin, may be added to the initial regimen for complication management or if antimicrobial resistance is suspected or proven (Freifeld et al., 2011). Ceftazidime is one of the third generation cephalosporin with antimicrobial coverage of gram negative organisms including *Pseudomonas aeruginosa* (Hess et al., 1998). Combination therapy of ceftazidime plus amikacin (Ceftaz/Amikacin) has proved to be effective and widely used in oncology patients with FN. Nevertheless, additional aminoglycosides are associated with an increased risk of nephrotoxicity and costs of treatment including an increased workload of the nursing staff. Therefore, the availability of broad-spectrum antibiotic monotherapy for oncology patients with FN would be useful for FN management (Hess et al., 1998).

Hess et al., (1998) performed a randomized trial to compare the efficacy between PIP/TZO monotherapy and combination therapy with Ceftaz/Amikacin in adolescents and adults cancer patients with FN. They concluded that treatment outcomes were not different. Meanwhile, Gorschlüter et al., (2003) reported that PIP/TZO was more effective than ceftriaxone plus gentamicin in febrile neutropenic treatment in adult patients. Randomized controlled trial in Turkey and Japan were conducted in pediatric oncology patients to compare the efficacy and safety between PIP/TZO and fourth generation cephalosporin and reported that no different outcomes between treatment groups (Ichikawa et al., 2011; Corapcioglu et al., 2006; Uygun et al., 2009). However, limited data between PIP/TZO monotherapy compared with Ceftaz/Amikacin therapy in pediatric oncology patients. The objectives of this study were to compare the efficacy of PIP/TZO with Ceftaz/Amikacin as an initial empirical treatment and to determine the microbial characteristics, antibiotics sensitivity and treatment outcomes of documented infections in pediatric oncology patients with FN. 

## Materials and Methods

*Patient eligibility*

Pediatric oncology patients aged < 18 years diagnosed with FN at Chiang Mai University Hospital were eligible for randomization. The diagnostic criteria for FN were fever > 38.3^o^C or fever > 38^o^C sustained for 1 hour plus neutropenia (absolute neutrophil count; ANC < 500 cells/cu.mm or < 1,000 cells/cu.mm with a predicted reduction to less than 500 cells/cu.mm in next 48 hours) (Freifeld et al., 2011). Patients who had HIV infection were excluded. Randomization exclusion criteria were septic shock, systemic intravenous antibiotic pretreatment within 5 days before randomization, history of hypersensitivity to any study drugs, fever associated with blood component transfusion and renal function impairment. The characteristics, antimicrobial sensitivity and treatment outcomes of documented infections including bloodstream infection and invasive fungal infection in all patients with randomized exclusion criteria were collected. According to previous study (Yildirim et al., 2008), sample size was calculated for non-inferiority or superiority trial for binary data, with alpha = 0.05 and 80% power. Non-inferiority or superiority margin = 0.2. Total 118 febrile neutropenic episodes were required, 59 episodes in each regimen.

*Randomization procedure*

This randomized, open-labeled, prospective controlled study was conducted between June 2017 and October 2018. To compare the efficacy of PIP/TZO with Ceftaz/Amikacin, patients were enrolled by the physicians attending the Pediatric Oncology unit at Chiang Mai University Hospital. The patients were randomly assigned (1:1) to the PIP/TZO (regimen A) group or Ceftaz/Amikacin (regimen B) group. Treatment was allocated by a computer-generated list in blocks of four. The assigned treatment regimens were sealed in opaque envelopes. Patients, investigators and the study team were not blinded to the study treatments. The study protocol was approved by institutional ethic committee. All patients and their parents gave written informed consent. 

*Therapeutic regimens*

Patients were randomized to receive either intravenous PIP/TZO, 320 mg/kg/day of piperacillin, divided every 8 hours (regimen A) or intravenous ceftazidime 100 mg/kg/day divided every 8 hours plus amikacin 15 mg/kg/day once daily (regimen B). If patients had localizing infections such as mucositis, skin infection or diarrhea, additional antibiotics were given. A re-evaluation was done at 48 hours after initial empirical treatment. If fever was persisted without documented localizing infection, meropenem 60 mg/kg/day divided every 8 hours was given. If the patient had methicillin-resistant *Staphylococcus aureus *(MRSA) infection, central venous catheter insertion or severe skin infection with shock, additional vancomycin 40 mg/kg/day divided every 6 hours was given. If the fever persisted after changing to meropenem with or without vancomycin for 48 hours, clinical evaluation for fungal infection was done and amphotericin B 1-1.5 mg/kg/day was given. When fever subsided, antimicrobial therapy will be continued for at least 7 days until the recovery of ANC (defined as ANC ≥ 500 cells/cu.mm). 

*Clinical and laboratory evaluation*

Clinical and demographic data were retrospectively reviewed. Complete history taking and physical examination were performed. The patients were investigated for complete blood count (CBC), blood urea nitrogen (BUN), creatinine (Cr), liver function test (LFT), C-reactive protein (CRP), urine examination and culture. Blood cultures, two specimens from peripheral sites, were performed at initial enrollment and next 24 hours. If patients had documented localizing infection, further investigations were performed according to clinical presentations.

*Definition of response*

The patients were evaluated every 48 hours until completion of the study treatment. The treatment was considered as early response when defervescence was observed in 48 hours. If the patients recovered from fever within 48 hours with disappearance of localizing infection and without modifications of initial empirical treatment, this was considered as complete response. Treatment failure was defined as persistent fever more than 48 hours, recurrent infection within 1 week after discontinuation of antimicrobial therapy, modification of initial treatment protocol or death from infections.

*Statistical analysis*

The analysis was performed with intention to treat principle. Data were analyzed by SPSS statistics version 17.0. Descriptive data were reported as median and quartile or frequency and percentage. To compare different parameters between the two groups, categorical variables were analyzed by Chi-square test or Fisher’s Exact test and continuous variables were analyzed by Student’s t-test or Mann Whitney U test. The *p-value* ≤ 0.05 was considered statistically significant. 

## Results

From June 2017 to October 2018, 118 febrile neutropenic episodes in 70 pediatric oncology patients were enrolled and all episodes in randomization group were included in statistical analysis. The median age was 7 (3-10) years. Forty-two (60%) patients were male. Five patients were in randomization exclusion criteria group. Clinical characteristics of febrile neutropenic episodes in 2 treatment groups were shown in Table 1.

**Table 1:** Clinical Characteristics of Febrile Neutropenic Episodes in the Two Treatment Groups

Clinical and hematologic parameters	PIP/TZO (n=59)	Ceftaz/Amikacin (n=59)	*p-value*
Age (year)	5 (3-10)	7 (5-10)	0.215
Gender: male	33 (55.9%)	35 (59.3%)	0.709
Weight (kg)	17.5 (12.3-28.3)	19.3 (15.0-35.0)	0.074
Peak temperature (◦C)	38.9 (38.5-39.3)	38.8 (38.4-39.2)	0.724
Time to needle (minute)	30 (30-45)	30 (30-55)	0.697
Hemoglobin (g/dl)	8.8 (8.0-10.3)	8.8 (7.7-9.8)	0.636
ANC (cells/cu.mm)	129 (19-453)	115 (10-582)	0.802
Platelet (x 1,000 cells/cu.mm)	78 (26-181)	54 (20-161)	0.301
CRP (mg/L)	20.3 (11.0-51.2)	18.4 (9.67-40.3)	0.572
Oral antibiotic prophylaxis	39 (66.1%)	40 (67.8%)	0.845
Oral antifungal prophylaxis	13 (22.0%)	8 (13.6%)	0.229
Underlying diseases			
Acute lymphoblastic leukemia	26 (44.1%)	29 (49.2%)	
Acute myeloid leukemia	5 (8.5%)	5 (8.5%)	
Neuroblastoma	5 (8.5%)	4 (6.8%)	
Lymphoma	4 (6.8%)	4 (6.8%)	
Rhabdomyosarcoma	4 (6.8%)	4 (6.8%)	0.923

PIP/TZO, Piperacillin/tazobactam; Ceftaz/Amikin, Ceftazidime plus amikacin; kg, kilogram; ◦C, degree Celsius; ANC, absolute neutrophil count; CRP, C-reactive protein

**Table 2:** Treatment Responses and Clinical Outcomes in 2 Treatment Groups

Treatment responses	PIP/TZO (n=59)	Ceftaz/Amikacin (n=59)	p-value
Early response to initial antibiotics	48 (81.4%)	40 (67.8%)	0.091
Complete response to initial antibiotics	41 (69.5%)	33 (55.9%)	0.128
Duration of fever (days)*	2 (1-4)	2 (1-4)	0.426
Duration of antibiotics (days)*	7 (7-10)	9 (7-12)	0.125
Duration of neutropenia (days)*	7 (5-9)	7 (5-11)	0.351
Treatment modification	18 (30.5%)	26 (44.1%)	0.128

*Data are median (interquartile range)

**Table 3:** Microbiologically Documented Infections in 2 Treatment Groups

Microbiologically documented infection (MDI)	PIP/TZO (n=59)	Ceftaz/Amikacin (n=59)	Excluded (n=5)
Hemoculture	3 (5.1%)	3 (5.1%)	4 (80.0%)
-*Staphylococcus epidermidis*	1 (1.7%)	1 (1.7%)	1 (20.0%)
-*Escherichia coli*	-	1 (1.7%)	-
-*Klebsiella pneumoniae*	-	1 (1.7%)	-
-*Pseudomonas aeruginosa*	1 (1.7%)	-	1 (20.0%)
-*Enterobacter cloacae*	1 (1.7%)	-	-
-*Morganella morganii*	-	-	1 (20.0%)
-*Candida tropicalis*	-	-	1 (20.0%)
Urine culture	6 (10.2%)	5 (8.5%)	-
-*Escherichia coli*	3 (5.1%)	-	-
-*Enterococcus faecalis*	-	1 (1.7%)	-
-*Enterococcus faecium *	2 (3.4%)	1 (1.7%)	-
-Others*	1 (1.7%)	3 (5.1%)	-

*Others are 1 *Proteus mirabilis*; 1 *Klebsiella pneumoniae*; 1 coagulase-negative Staphylococci and 1 *Staphylococcus haemolyticus*

Response to treatment and clinical outcomes were shown in Table 2. Early response, complete response to initial treatment, duration of fever, duration of antibiotics, duration of neutropenia and treatment modification were not statistically different between two groups. This study found that 35/59 (59.3%) episodes in PIP/TZO group and 30/59 (50.8%) episodes in Ceftaz/Amikacin group had FN without documented infection. Meanwhile, the most common clinically documented infection (CDI) was oral mucositis, 11/59 (18.6%) episodes in PIP/TZO group and 14/59 (23.7%) episodes in Ceftaz/Amikacin group. None of the patients in our study had central line insertion, therefore two specimens of blood culture from peripheral sites were performed at initial enrollment and next 24 hours. The bloodstream infections were documented in 3/59 (5.1%) episodes in each group. Gram-negative bacteremia was more common than gram-positive bacteremia. Urinary tract infection (UTI) was diagnosed when single pathogenic organism > 10^5^ colony forming unit (cfu)/ml was identified from urine culture. Urinary tract infections were documented in 6/59 (10.2%) episodes in PIP/TZO group and 5/59 (8.5%) episodes in Ceftaz/Amikacin group. Microbiologically documented infections were shown in Table 3. Three patients in this study received additional vancomycin. One patient in Ceftaz/Amikacin group received vancomycin as initial treatment due to history of *Enterococcus faecium* UTI (sensitive to vancomycin). The other two patients, one in each randomization group, had gram positive bacteremia. Therefore vancomycin was given on the next day of randomization but not as initial treatment. The antimicrobial sensitivity for bloodstream infections were as following: *Staphylococcus epidermidis* was sensitive to vancomycin, *Escherichia coli*, *Klebsiella pneumoniae*, *Pseudomonas aeruginosa* and *Enterobacter cloacae* were all sensitive to ceftazidime, meropenem and PIP/TZO. Invasive pulmonary aspergillosis (IPA) was diagnosed in 4 patients in PIP/TZO group and 2 patients in Ceftaz/Amikacin group.

Four bloodstream infections were documented in 5 patients in randomization exclusion criteria group. *Morganella morganii* and *Pseudomonas aeruginosa *bacteremia were sensitive to ceftazidime, meropenem and PIP/TZO. *Staphylococcus epidermidis* was sensitive to vancomycin and candidemia (*Candida tropicalis*) was documented in one patient.

During the study period, no severe adverse events were observed in 2 treatment groups. All patients in randomization survived. Two in 5 patients in randomization exclusion criteria group died due to bloodstream infections from *Morganella morganii* and *Staphylococcus epidermidis*.

## Discussion

This study demonstrated that both PIP/TZO monotherapy and combination therapy with Ceftaz/Amikacin was effective and safe for the empirical treatment in pediatric oncology patients with FN. Early response and complete response to initial antibiotics, duration of fever, duration of antibiotics, duration of neutropenia and treatment modification were not significantly different between the two groups. The results were comparable with previous studies comparing the efficacy between PIP/TZO and fourth generation cephalosporin in pediatric oncology patients with FN. Corapcioglu et al., (2006) and Uygun et al., (2009) demonstrated that PIP/TZO and cefepime were both effective and safe for treatment FN in pediatric patients with cancer. Ichikawa et al., (2011) reported PIP/TZO and cefozopran were both effective and safe. Similarly, a study in adolescents and adult oncology patients reported no different outcomes between PIP/TZO and Ceftaz/Amikacin (Hess et al., 1998). On the other hand, a study in adult hematologic malignancy patients showed that PIP/TZO was more effective and cost-efficient than ceftriaxone plus gentamicin (*p-value *0.0047) (Gorschlüter et al., 2003). 

Corapcioglu et al., (2006) showed that the total cost of treatment of FN per episode which included the cost of antimicrobial therapy, hospitalization, supportive care and daily cost were not different between PIP/TZO group and cefepime group in pediatric oncology patients with FN. Furthermore, one study in adult oncology patients with FN reported the lower cost in PIP/TZO monotherapy (Hazel et al., 1997). In our country, the cost of PIP/TZO is higher than Ceftaz/Amikacin, but the other direct and indirect costs associated with both treatments were not analyzed, so we were not able to compare the cost of treatment between the two groups.

Most of our patients had fever without documented infections which were similar to a study from Turkey (Corapcioglu et al., 2006; Uygun et al., 2009). In the patients with documented infections, gram-negative bacteremia was more frequently observed not only in randomization group but also in the excluded group. For gram-positive organisms, only *Staphylococcus epidermidis *was documented. These findings were different to the studies by Hess et al., (1998), Ichikawa et al., (2011) and Corapcioglu et al., (2006) that reported the high prevalence of gram-positive bloodstream infection. The difference may largely result from the use of central venous catheter. In our institute, central venous catheter insertion was not commonly used. For antimicrobial susceptibility data, our study showed that all gram-negative organisms had no resistance pattern. 

This study demonstrated that the treatment response were not statistically different between the PIP/TZO monotherapy and combination therapy with Ceftaz/Amikacin. Also, no severe adverse effects were observed in both treatment groups. We conclude that both PIP/TZO monotherapy and combination therapy with Ceftaz/Amikacin are effective for pediatric oncology patients with FN. 
